# Hospital Festivals and Meetings

**Published:** 1894-11-24

**Authors:** 


### HOSPITAL FESTIVALS AND MEETINGS.

### EPILEPTIC COLONY AT CHALFONT ST. PETERS.

On Wednesday, November 14th, the foundation-stone of the first permanent home of this newly-established colony was laid by Mr. Passmore Edwards, through whose generosity the present site has been secured.

The colony has been started through the medium of the National Society for the Employment of Epileptics, whose scheme was first laid before the public in January, 1893, at a fully-attended meeting at the Mansion House, amongst its warm supporters being the late Sir Andrew Clark and many other well-known medical men. The plan to be followed was "to provide a home for those necessitous epileptics who are able and willing to work, but for whom their friends are unable to procure employment on account of the infliction which bars their admission into ordinary fields of industry. It is intended that cottages should be arranged for these, and should each accommodate, according to their size, from ten to twenty epileptics. The sexes would be separated and the children from the adults. Market gardening, spade and barrow labour, cow-keeping, dairy work, and poultry farming would be the first industries, and later on would follow boot-making, carpentering, bookbinding, printing, and other industries, and for the women laundry work, sewing, cooking, and various domestic services. All would be employed in the manner best suited to their condition." The poor are to receive the first consideration, but it is contemplated to extend the benefits of the institution to those who can afford adequate payment as boarders.

Part of this scheme is now an accomplished fact. The farm ultimately bought, after much consideration, is situated in Buckinghamshire, at Chalfont St. Peters, and consists of about 130 acres. It is a healthy and beautiful spot, and in every way suitable for its purpose. Temporary iron buildings have here been erected, and the first epileptics were received in August last, now numbering some 14 or 15.

It is not intended to have one large institution, but a number of smaller buildings, each to contain 18 to 20 epileptics, with the necessary staff. The present iron building accommodates 18 patients, the matron, two male attendants, and servants; the arrangements throughout appeared most comfortable and efficient, and Miss Delaney, the matron, is much to be congratulated on the order and happiness which evidently reigns in the colony. The first permanent building, of which the stone was laid last week, has been designed by Mr. Keith D. Young.

It was very unfortunate that the day should have been hopelessly wet and stormy, but in spite of the persistent rain a fair number of visitors, all evidently keenly interested in the experiment, found their way to Chorley Wood Station last week. The farm is a four-mile drive from there, but plenty of carriages were in waiting and every arrangement had been made for the comfort of the visitors. On account of the weather the short dedicatory service was read in the dining-room of the present building by the Rev. F. Woods, the vicar of the parish, after which the stone was duly laid by Mr. Passmore Edwards, and tea was afterwards provided in the dining-room, which was prettily decorated and looked bright and cheerful. Mr. Micholls, the chairman of the executive committee, drew a hopeful picture of the future of the colony in a short speech, and Dr. Buzzard gave an encouraging account of the good results already experienced in the greatly-improved health of the colonists. Amongst other friends of the movement who were present were Dr. Ferrier, Dr. Savage, Dr. Fletcher Beach, and Dr. Colman. A warm vote of thanks to Mr. Passmore Edwards for his generous support was unanimously given.

It is much to be hoped that now a beginning has been made and it is possible for the public to see what is being done that a hearty measure of support will be accorded to this imperatively needed society. Many things, too, in kind as well as in money are wanted in the colony to help to make it homelike and comfortable. Books, papers, magazines, games, music, tools and implements, plants and seeds are asked for. Will not those who have abundance of such things sometimes remember what pleasure may be given could some of their store be sent to the Epileptic Colony at Chalfont St. Peters?

